# Myocardial Infarction Simulated From Improper Telemetry (MISFIT): An Autobiographical Case Report

**DOI:** 10.7759/cureus.53197

**Published:** 2024-01-29

**Authors:** Philip R Cohen, Brent M Gudenkauf

**Affiliations:** 1 Dermatology, University of California, Davis Medical Center, Sacramento, USA; 2 Dermatology, Touro University California College of Osteopathic Medicine, Vallejo, USA; 3 Cardiology, The Texas Heart Institute, Houston, USA

**Keywords:** telemetry, myocardial, misplacement, misfit, misdiagnosis, lead, infarction, heart, cardiac, attack

## Abstract

An electrocardiogram, used to not only assess the rate and rhythm of the heart but also to evaluate for injury to the heart, is performed by attaching 12 leads to the patient’s body. A myocardial infarction can be mimicked by the misplacement of the leads. A 58-year-old man with long-distance running-associated bradycardia developed postoperative atrial fibrillation with a rapid ventricular response. He converted to normal sinus rhythm after a single oral dose of 30 milligrams of diltiazem; however, the automated reading of the electrocardiogram performed in the hospital showed new changes suggestive of a postero-lateral myocardial infarction, including Q waves in leads I and aVL, as well as early precordial R wave progression with R waves and positive T waves in V_2_ and V_3_, and a dominant R wave (R wave to S wave ratio greater than one) in V_2_. A cardiac work-up was entirely normal: serial troponin levels, thyroid stimulating hormone, echocardiogram, computerized tomography of the chest, and Doppler studies of the extremities. Lead misplacement during the electrocardiogram was suspected during the subsequent evaluation by an astute cardiologist; the findings were diagnostic for a left arm to right arm limb lead reversal. All the changes in myocardial infarction were absent when the electrocardiogram was repeated in the office. Misplacement of leads during an electrocardiogram is not a rare event; therefore, the clinician needs to consider the possibility of improper placement of the leads when evaluating an electrocardiogram. Indeed, emotional distress, additional diagnostic procedures, and potentially harmful procedures may be experienced by the patient from incorrect diagnoses based on electrode misplacement during an electrocardiogram; in addition, there are often increased costs to the patient and the healthcare system. Therefore, in the setting of an incorrect diagnosis attributed to lead misplacement during the performance of an electrocardiogram, the acronym MISFIT (which uses the first letters of the words “myocardial infarction simulated from improper telemetry”) has been introduced. In conclusion, it is important to emphasize that a MISFIT is characterized by an electrocardiogram ‘mis’diagnosis of a myocardial infarction that does not ‘fit’ with the clinical scenario.

## Introduction

An electrocardiogram evaluates the electrical conduction patterns of the heart. It is used to assess not only the heart rate and rhythm but also for injury to the heart. After an acute myocardial infarction, it can be the earliest objective indicator of heart injury [1-3].

The results of an electrocardiogram are based on the correct placement of the 12 leads that are applied to the patient’s body. A non-intentional error caused by the improper placement of one or more recording electrodes on the skin can result in an inaccurate evaluation of the individual being assessed. For example, misplacement of the leads can mimic a myocardial infarction [1,4-9].

A 58-year-old man who had recently had extensive lower back surgery developed postoperative atrial fibrillation on the third postoperative day; the rate was controlled with a single oral dose of diltiazem 30 mg. After his episode of atrial fibrillation, the electrocardiogram reported a postero-lateral myocardial infarction. A subsequent review of the electrocardiogram showed that the observed electrocardiogram changes were a myocardial infarction simulated from improper telemetry (MISFIT). Salient features that can prompt clinicians to consider the possibility of electrocardiogram electrode misplacement are summarized.

## Case presentation

A healthy 57-year-old man, who previously had completed 20 marathons in Houston, presented with bilateral sciatica symptoms. His magnetic resonance imaging of the lower back showed severe bilateral spinal canal stenosis from the second lumbar vertebrae to the fifth lumbar vertebrae. On two occasions, he received computed tomography-guided epidural corticosteroid injections localized to the site of the most severely affected nerves; each injection provided pain relief, and he was able to continue running half marathons.

**Figure 1:**
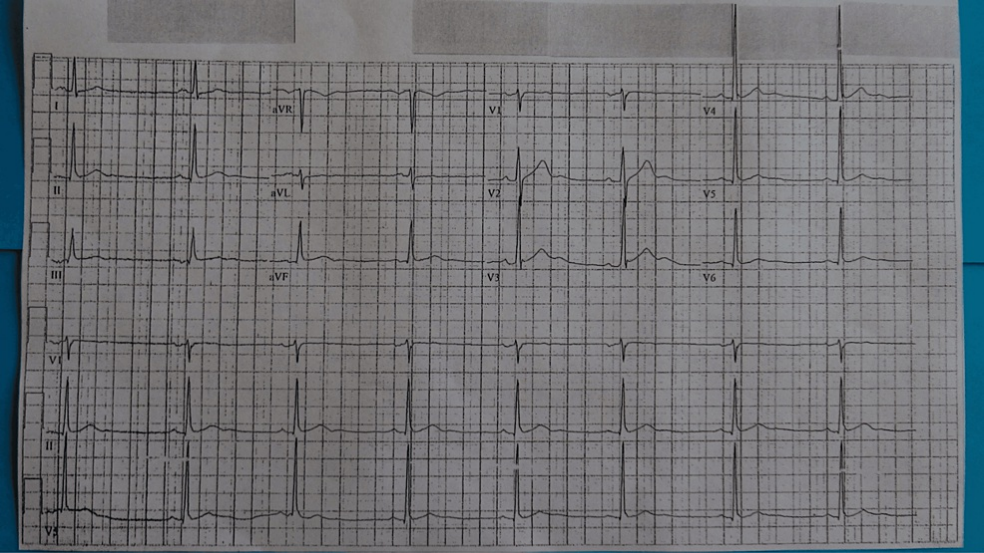
Preoperative electrocardiogram with sinus bradycardia and no evidence of myocardial infarction A 58-year-old man with no history of heart disease had a preoperative electrocardiogram prior to his spinal surgery. He was a long-distance runner and had completed a yearly marathon in 20 of the prior 26 years; during the 26 years, he had also completed several half-marathons. The electrocardiogram showed sinus bradycardia of 47 beats per minute, attributed to his running, and some nonspecific ST and T wave changes.

Within 18 months from the initial presentation, he continued to experience progressive right leg weakness. Subsequently, he developed left leg weakness and could not stand up on the toes of either foot. He was 58 years old when he decided to have surgery; his preoperative electrocardiogram showed sinus bradycardia of 47 beats per minute and some nonspecific ST and T wave abnormality (Figure 1).

His spinal surgery consisted of a decompression laminectomy without fusion at age 58. A surgical complication included a dural sac tear; repair was attempted during the operation. Postoperatively, he had to remain lying on his back in the hospital bed for the next 48 hours. The dural sac tear had not been adequately treated intraoperatively; a cerebrospinal fluid leak developed that was not discovered until after his discharge from the hospital [10].

During the evening of his third postoperative day, after he had been allowed to ambulate, he became progressively short of breath. The nurse had increased the oxygen flow to his nasal cannula to four liters; despite the increased oxygen he was receiving, his oxygen saturation was only 91 percent. He was becoming confused and had a new tremor in his hands.

He was not short of breath; his respiratory rate was 20 breaths per minute. His systolic and diastolic blood pressures were 100 millimeters mercury and 80 millimeters mercury, respectively. However, his pulse was rapid and irregular. An electrocardiogram revealed atrial fibrillation with a rapid ventricular response; his pulse was 137 beats per minute.

He was seen by the cardiology fellow and was given a single oral dose of 30 milligrams of diltiazem. Within the next six hours, his heart rate decreased to less than 100 beats per minute and did not become that rapid again. Therefore, he did not receive any additional diltiazem.

The initial impression of the cardiologist was that he had experienced idiopathic postoperative atrial fibrillation. To rule out other possible etiologies for the new onset of atrial fibrillation, he underwent a cardiac evaluation. His thyroid stimulating hormone was normal, and his serial troponin levels were negative.

The computerized tomography of the chest was normal to rule out a pulmonary embolus, and Doppler studies of the upper and lower extremity deep veins were negative for thrombosis. The echocardiogram was normal; not only the left and right ventricle size and function were normal, but also the size of both the left atrium (volume index, 23.1 ml/m^2^) and the right atrium were normal. In addition, the mitral valve was normal in structure, with trace mitral valve regurgitation, and there was no evidence of pericardial effusion.

**Figure 2:**
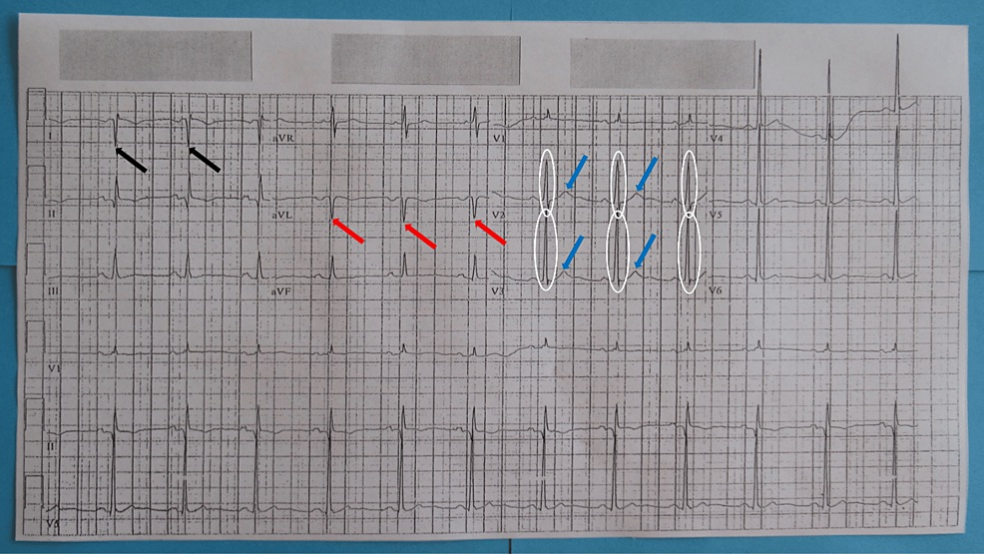
Electrocardiogram showing new changes suggestive of a postero-lateral myocardial infarct After experiencing paroxysmal postoperative atrial fibrillation, the 58-year-old man’s electrocardiogram performed in the hospital showed normal sinus rhythm; however, new changes were observed that the automated reading of the electrocardiogram which was diagnosed as suggestive of a postero-lateral myocardial infarction. There were Q waves in leads I (black arrows) and lead aVL (red arrows). In addition to early precordial R wave progression with R waves, there were positive T waves in V_2_ and V_3_ (blue arrows). Also, in V_2_ and V_3_, a dominant R wave resulted in the R wave to S wave ratio being greater than one (white ovals).

Within two days, he had spontaneously converted into normal sinus rhythm; however, an automated reading of his electrocardiogram interpreted that the new changes were suggestive of a postero-lateral infarct, with Q waves in lead I and lead aVL, as well as early precordial R wave progression with R waves and positive T waves in V_2_ and V_3_, and a dominant R wave (R wave to S wave ratio greater than one) in V_2_ and V_3_ (Figure 2).

Both he and his wife (who is a physician) were concerned about this finding; they were provided no explanation. However, he was discharged to home the following day.

He was seen by a senior cardiology attending three weeks later. His respiratory rate was 18 breaths per minute, and his systolic and diastolic blood pressure were 139 millimeters mercury and 78 millimeters mercury, respectively. However, his pulse was 66 beats per minute and regular. Both he and his wife asked the doctor about the abnormal electrocardiogram that was done in the hospital. The cardiologist suspected that the leads had been misplaced during the electrocardiogram.

**Figure 3:**
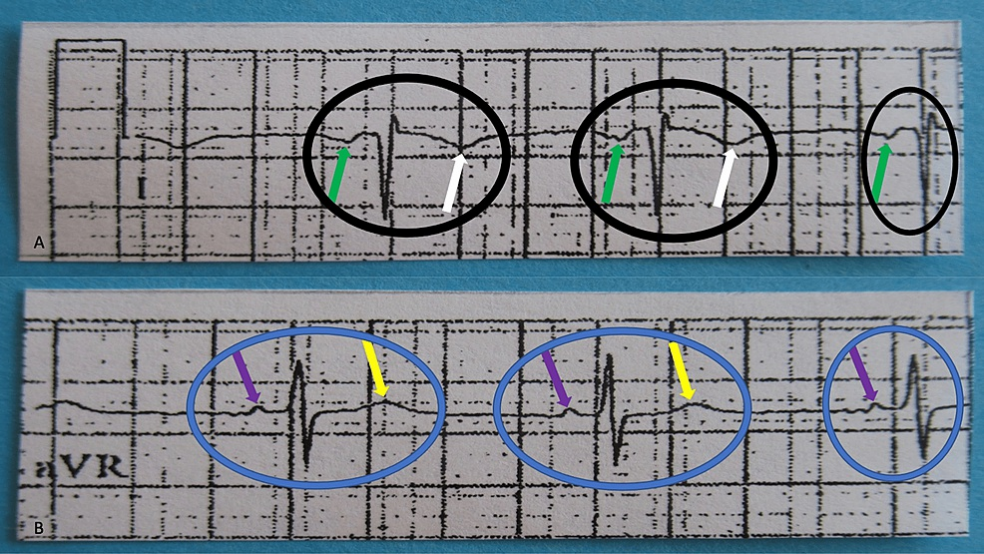
A closer view of lead I and lead aVR of the 58-year-old man’s normal sinus rhythm electrocardiogram in which the automated reading misdiagnosed a postero-lateral myocardial infarction caused by a reversal of the upper extremity limb leads There are several salient features that establish that the left arm and right arm limb leads were reversed, which is best demonstrated by the findings in lead I. This lead represents the voltage difference between the electrodes placed on the left and right arms. Lead I (shown in A) is entirely negative (black oval); the P wave is negative (green arrows); the QRS complex has a negative deflection; and the T wave is negative (white arrows). In contrast, lead aVR (shown in B) has become positive (blue oval): the P wave is positive (purple arrows), the QRS complex has a positive deflection, and the T wave is positive (yellow arrows). In summary, the features suggestive of limb lead reversal are the positive P waves (purple arrows) in aVR (shown in B) and the negative P waves (green arrows) in lead I (shown in A); the highly unlikely possibility of a highly ectopic atrial focus originating from the left atrium, with the positive deflection (purple arrows) in lead aVR (shown in B) and the negative deflection (green arrows) in lead I (shown in A) helps establish the diagnosis that the limb leads have been reversed.

Indeed, the patient’s electrocardiogram showed left arm to right arm limb lead reversal, which is best demonstrated by the findings in lead I (Figure 3).

This lead represents the voltage difference between the electrodes placed on the left and right arms. By reversing these leads, the tracing recorded represented the voltage difference between the right arm and the left arm and became inverted, with the vector directed towards the right arm. In a patient without dextrocardia, the electromotive forces of the heart move toward the left side of the body and thus in the opposite direction from this newly recorded vector, causing negative deflections. An astute observer can appreciate that lead I (including the P wave, the QRS complex, and the T wave) is entirely negative in this electrocardiogram.

In contrast, lead aVR has become positive (Figure 3). With limb lead reversal, this lead’s vector is now directed toward the left arm, which is the predominant direction of the electrical current.

Another feature suggestive of limb lead reversal is the positive P waves in lead aVR and the negative P waves in lead I (Figure 3). Assuming no limb lead reversal, this would suggest a highly ectopic atrial focus, such as one originating from the left atrium; in this setting, electricity would first travel towards the right, yielding a positive deflection in lead aVR and a negative deflection in lead I (Figure 3). The ectopic atrial rhythm would be a rare diagnosis to make *de novo*.

**Figure 4:**
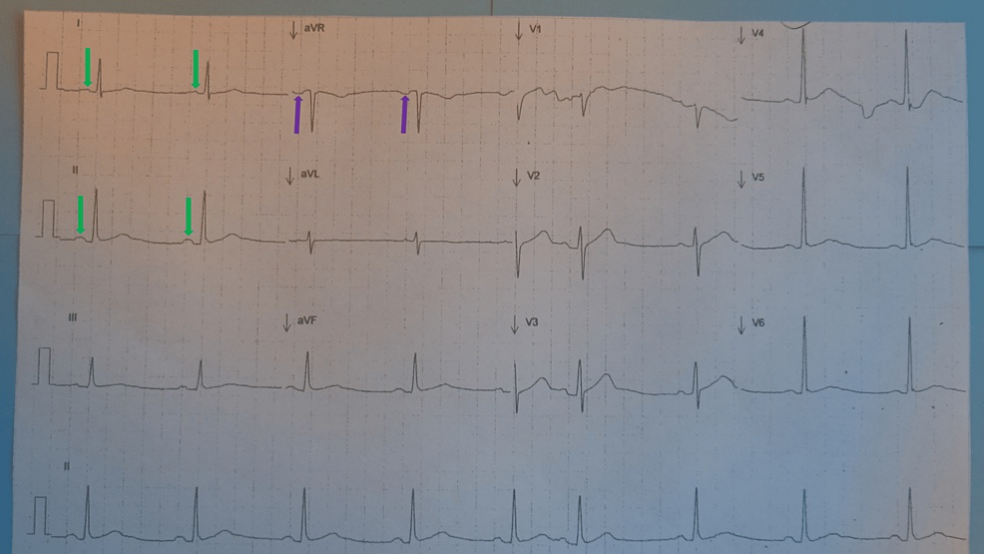
The 58-year-old man's repeat electrocardiogram without myocardial infarction changes All the changes of myocardial infarction were absent on an electrocardiogram subsequently performed at the cardiologist’s office. No Q waves were present. Normal R wave progression of the precordial leads was observed. In addition, normal P wave morphology was seen, with positive P waves (green arrows) present in leads I and II, and negative P waves (purple arrows) present in lead aVR.

There were no changes in myocardial infarction on an electrocardiogram subsequently performed at the office visit with the cardiologist (Figure 4).

There were no Q waves present. In addition, normal R wave progression of the precordial leads was now observed. The P wave morphology was now normal; the P waves were not only positive in lead I and lead II but also negative in lead aVR.

## Discussion

Classic and characteristic changes, such as ST-segment elevation and Q waves, may be observed on an electrocardiogram that demonstrates either an acute or previous myocardial infarction. A lateral myocardial infarction is diagnosed when these changes appear in leads I and aVL. In contrast, an inferior wall myocardial infarction shows changes in leads II, III, and aVF [9].

The results of an electrocardiogram are only as valid as the accuracy of the placement of the 12 leads. The four limb electrodes are placed distally on the extremities; the right leg electrode is a ground wire [6]. The electrical signals may be distorted if the upper-extremity leads are placed on the radial pulse [11].

The precordial leads are placed on the right side of the chest, the left side of the chest, and the left flank. Surface anatomy landmarks can be used to determine the proper placement of the precordial electrodes (Table 1) [3,6,12,13].

**Table 1:** Appropriate location of precordial electrode leads ^a^The sternal angle (angle of Louis) can be used to aid in the detection of the second intercostal space; the second intercostal space can be located by palpating from the sternal angle. The sternal angle is approximately five centimeters below the sternal notch; it is a bony ridge that represents the junction of the manubrium and the body of the sternum. The second intercostal space is immediately lateral and just inferior to the sternal angle. The second intercostal space is two spaces above the fourth intercostal space (the location of V_1 _and V_2_) and three spaces above the fifth intercostal space (the location of V_4_, V_5_, and V_6_). ^b^The fourth intercostal space can also be located by using the medial left clavicle. The first rib lies beneath the medial clavicle; below the first rib is the first intercostal space, which is three spaces above the fourth intercostal space (the location of V_1_ and V_2_). Ref: references

Precordial electrode	Anatomic location (x-axis)	Anatomic level (y-axis)	Ref
V_1_	In the fourth intercostal space^a,b^	Along the right margin of the sternum	[3,6,12,13]
V_2_	In the fourth intercostal space^a,b^	Along the left margin of the sternum	[3,6,12,13]
V_3_	It is placed midway between V_2_ and V_4_	Midway along a straight line between V_2_ and V_4_	[3,6,12,13]
V_4_	In the fifth intercostal space; in women, the electrode should be placed under the breast fold	In the left mid-clavicular line	[3,6,12,13]
V_5_	In the fifth intercostal space and at the same horizontal level as V_4_; in women, the electrode should be placed under the breast fold	In the left anterior axillary line	[3,6,12,13]
V_6_	In the fifth intercostal space and at the same horizontal level as V_4_	In the left mid-axillary line	[3,6,12,13]

Upward displacement of leads V_1_ and V_2_ above the fourth intercostal space is the most frequent incorrect positioning of the precordial electrodes [3,12-15].

When evaluating any electrocardiogram, the clinician needs to consider the possibility of misplacement of the electrocardiogram leads, especially in a patient with no known cardiac disorder [12]. Some of the alterations in the normal electrocardiogram pattern caused by common lead reversals and misplacements are summarized in Table 2 [5,6,9,16].

**Table 2:** Lead reversals, false clinical conditions, and electrocardiogram patterns ^a^Reversal of the left arm and the right arm is the most common lead misplacement. ^b^In lead V_1_, when the R wave to S wave ratio (R/S ratio) is equal to or greater than one, the prominent R wave in lead V_1_ is referred to as a tall lead V_1_ or a tall RV_1_. In addition to misplaced precordial leads, the differential diagnosis of tall R waves in lead V_1_ includes acute right ventricular dilation (acute right heart strain), dextrocardia, hypertrophic cardiomyopathy, left ventricular ectopy, normal variant, posterior myocardial infarction, progressive muscular dystrophy, right bundle branch block, right ventricular hypertrophy, and Wolff-Parkinson-White syndrome (Type A) [16]. Ref: references

Lead reversal	Conditions mimicked clinically	Presenting electrocardiogram pattern	Ref
Left arm and right arm^a^	Old lateral myocardial infarction; non-sinus atrial rhythm	Large Q-wave in I and aVL; inverted P waves in II, III, and aVF; leads I and aVL appear as the inverse of V_5_ -V_6_	[5,6,9]
Left leg and right arm	Old inferior wall myocardial infarction; non-sinus atrial rhythm	Large Q-waves in II, III, and aVF; inverted P waves in II, II, and aVF	[9]
Right arm and right leg	Diffuse low voltage-associated conditions	All limb leads show diffuse low voltage, especially lead II (there is a near-flat line appearance); junctional rhythm or non-sinus atrial rhythm	[6,9]
Precordial leads	Posterolateral myocardial infarction; mirror-image dextrocardia	V_1_-V_6: _break in normal R-wave progression^b^	[6,9,16]
Left arm and left leg	None	Lead III: insignificant Q-wave	[9]
Left arm and right leg	None	No change	[9]
Left leg and right leg	None	Lead III: low voltage	[9]

A summary of some of the other alterations to the electrocardiogram pattern based on the placement of the precordial leads or associated with limb arterial pulsation is listed in Table 3 [1,11,17].

**Table 3:** Electrocardiogram changes suggestive of the possibility of misplaced peripheral electrodes Ref: references

Electrocardiogram findings suggestive of misplaced leads	Ref
Abnormal P wave orientation and abnormal P axis (positive P wave in aVR and negative P wave in I or II)	[17]
Abnormal QRS axis deviation (QRS axis over -120 degrees, positive R wave in aVR)	[17]
Bizarre appearance to electrocardiogram	[1,17]
Discordant relationship between aVR and V_6_	[17]
Isolated abnormal progression of the R wave in the precordial leads	[17]
Limb arterial pulsation-associated artifacts: the artifacts appear after the QRS complex	[11]
Limb arterial pulsation-associated artifacts: the artifact is recorded on the electrocardiogram tracing	[11]
Limb arterial pulsation-associated artifacts: when a single limb arterial pulsation is the source of the artifacts, only one limb lead (I, II, or III) will remain unaffected	[11]
Negative QRS complex with concordant negative T waves in several of leads I, II, III, and aVF	[17]
Poor R wave progression in the precordial leads that mimics old anterior or anteroseptal myocardial infarction	[17]
The amplitude of the QRS complex will gradually decrease from lead V_1_ to V_6 _when the electrodes for leads V_1_ to V_6_ are placed on the right side of the chest	[11]
Unexpected changes of QRS morphology or axis between electrocardiograms in the same individual	[17]
Very low amplitude of QRS complex in leads I, II, or III	[17]

Incorrect electrocardiogram diagnoses resulting from misplacement of the leads are not a rare event [1,3]. Several studies have documented this problem (Table 4) [2,14,15,18,19].

**Table 4:** Studies assessing incorrect electrocardiogram diagnoses resulting from electrode misplacement mV,: millivolts; Ref: references; <: less than; o: degrees

Year	Comments	Ref
1996	The study included 30 experienced technicians evaluating their placement of precordial electrodes. In more than 50 percent of routine applications of leads V_1 _and V_2_, there was superior quadrant displacement of more than 0.625 inch; this was an indication that these electrodes were commonly placed both high and wide of their anatomically defined precordial sites. Similarly, in 30 percent to 50 percent leads of routine application of V_4_ through V_6_, there was an inferior and leftward displacement of more than 0.625 inch; therefore, this indicates that these lateral precordial electrodes are commonly placed both low and wide of their respective anatomic sites.	[15]
2007	Investigators evaluated the incidence of lead misplacement in the cardiology outpatient clinic (739 electrocardiograms) and the intensive care unit (99 electrocardiograms). Morphologic changes suggestive of lead misplacement included: abnormal R progression in the precordial leads, negative P waves in lead I and /or II, positive P wave in lead aVR, QRS axis between 180^o^ and -90^o^, and very low (<0.1 mV) amplitude in an isolated peripheral lead. Based on these criteria, electrode misplacement was suspected in 37 electrocardiograms and confirmed in seven. The frequency of electrocardiogram artifact due to switched electrodes was 4.0 percent (4 electrocardiograms) at the intensive care unit and 0.4 percent (3 electrocardiograms) at the outpatient clinic. Therefore, the frequency of errors in electrocardiogram performance was significantly greater (P-value = 0.005) in an acute medical care setting.	[18]
2008	A total of 119 participants including 72 doctors (20 cardiologists and 52 non-cardiologists), 37 nurses, and 10 cardiac technicians were asked to mark on diagrams the positions of the precordial electrodes V_1_ to V_6_. The correct position of V_1_ was only identified by 90 percent of cardiac technicians, 49 percent of nurses, 31 percent of non-cardiologist physicians, and only 16 percent of cardiologists. In addition, V_5_ and V_6_ were also frequently mispositioned on the lateral chest wall.	[14]
2012	The investigators hypothesized that incorrect electrode placement has a reasonable chance of changing the diagnosis of an echocardiogram. The researchers found that displacing leads V_1_ and V_2_ in the second intercostal space (instead of the fourth intercostal space) also resulted in offsetting the placement of the other precordial leads. They observed that there was a 17 percent to 24 percent chance that the diagnostic interpretation of the electrocardiogram would be different based on the lead misplacement.	[19]
2020	The incidence and economic burden to healthcare of precordial lead mispositioning were evaluated. During a consecutive period of 12 months, 9,424 outpatient electrocardiograms were performed; 1,018 (10.8 percent) were determined to being possibly being falsely labeled as some type of myocardial infarction suggesting underlying coronary artery disease. The investigators concluded that the abnormal electrocardiograms resulted in false diagnoses and subsequent unnecessary cardiovascular testing with not only increased risk and cost to the patient but also increased financial burden to the national healthcare in other amounts of billions of dollars annually.	[2]

Importantly, the affected individual and their family, like the patient in this report, may be unnecessarily worried about the possibility of a myocardial infarction. Therefore, we have introduced the acronym MISFIT (which uses the first letters of the words “myocardial infarction simulated from improper telemetry”) to emphasize that the electrocardiogram ‘mis’diagnosis of a myocardial infarction does not ‘fit’ with the clinical scenario.

For example, the man in this report experienced paroxysmal atrial fibrillation in the postoperative period; two days later, after a single 30-milligram dose of diltiazem to control the rapid rate, he converted back to normal sinus rhythm. The electrocardiogram that documented his return to normal sinus rhythm had new changes suggestive of a postero-lateral infarct. However, additional evaluation of that electrocardiogram showed changes consistent with misplaced electrocardiogram leads to account for the misdiagnosis of myocardial infarction; the MISFIT occurred because of a left arm to right arm limb lead reversal. A subsequent electrocardiogram taken in the cardiologist’s office did not show any findings of myocardial infarction.

In addition, the incorrect placement of the electrodes can also result in a false negative diagnosis by displaying a benign-appearing electrocardiogram and thereby missing the actual cardiac problem of significance [8,13]. In a study of 10 volunteers, a baseline electrocardiogram was obtained and compared to an electrocardiogram after the precordial electrodes were moved. In one person whose baseline electrocardiogram demonstrated a prior inferior myocardial infarction, movement of all the precordial leads into one intercostal space superior to the standard position for each resulted in an electrocardiogram that was normal [13]. In a subsequent study that included three patients with electrocardiographic signs of inferior myocardial scars, normal electrocardiograms without signs of ischemia were obtained by placing the electrode of the left leg to the left arm [8].

Albeit rarely, misplacement of the electrodes has prompted additional investigation that has discovered an unsuspected cardiac abnormality [3]. However, more commonly, incorrect diagnoses based on electrode misplacement can be associated with increased costs to the patient and the healthcare system. These errors in lead misplacement frequently result in unnecessary additional diagnostic evaluations and potentially harmful therapeutic procedures [2,14].

The initial approach to the management of a MISFIT is to consider the possibility of lead misplacement [1,3,5,8]. "REVERSE" is a mnemonic that may help to identify lead reversal on a 12-lead electrocardiogram (Table 5) [4].

**Table 5:** Frequent causes of electrocardiographic electrode placement and artifacts: REVERSE mnemonic ^a^Also, negative P-QRS-T in lead I. And concordant negative QRS and T waves in lead I, II, III, aVL, and/or aVF (right arm and left leg, right arm and left arm, and right leg and left leg misplacement). ^b^Also negative R wave in lead I, positive R wave in aVF. ^c^An isolated “flat” lead. ^d^The P wave in lead I is greater than in lead II. The sensitivity of this finding is 90 percent. ^e^Predominant R wave in V_1_ and predominant S wave in V_6_. Also, the orientation of the QRS complexes in leads I and V_6_ should be the same. ^f^Negative P waves in lead I. ^g^These artifacts mimic tachycardias or ST-T changes. M: mnemonic; mV: millivolt; <: less than

M	Abnormal electrocardiogram finding	Reversal of electrode leads
R	R wave is positive in aVR (P wave also is positive).^a^	Left arm and right arm
E	Extreme axis deviation: QRS axis between +180 degrees and -90 degrees^b^	Left arm and right arm
V	Very low voltage (<0.1 mV) amplitude in an isolated limb lead^c^	Right leg and left arm or right arm
E	Exchange amplitude of the P waves^d^	Left arm and left leg
R	R wave: abnormal progression in the precordial leads^e^	Precordial leads (V_1_ through V_6_)
S	Suspect dextrocardia^f^	Left arm and right arm
E	Eliminate noise and interference^g^	Artifact

Technological advances in the instruments used for performing this task have begun to incorporate algorithms that enable machine learning techniques for detecting electrode misplacement and interchanges when recording electrocardiograms [19,20]. Once the diagnosis of inadequate placement of the electrocardiogram electrodes is suspected, particularly when the patient’s clinical findings are not suggestive of ischemic heart disease, the electrocardiogram should be repeated after confirming that all the leads have been correctly placed [1,4,6].

## Conclusions

Misplacement of the electrocardiogram leads can mimic a myocardial infarction. The preoperative electrocardiogram of a 58-year-old man showed bradycardia, attributed to long-distance running; he developed postoperative atrial fibrillation with a rapid ventricular response, which converted to normal sinus rhythm after a single oral dose of 30 milligrams of diltiazem. Subsequently, the automated reading on an electrocardiogram performed in the hospital showed new changes suggestive of a postero-lateral myocardial infarction. Lead misplacement during the electrocardiogram was suspected; diagnostic findings of a left-arm to right-arm limb lead reversal were observed. When the electrocardiogram was repeated in the office, all the changes of myocardial infarction were absent. Improper placement of electrocardiogram leads is not a rare event. The acronym MISFIT (which uses the first letters of the words “myocardial infarction simulated from improper telemetry”) may be applied when the automated reading of the electrocardiogram suggests a ‘mis’diagnosis of a myocardial infarction that does not ‘fit’ the clinical scenario and was caused by leads that were not placed correctly. In conclusion, the clinician needs to consider the possibility of improper placement of the leads when evaluating any electrocardiogram, especially in a patient with no known cardiac disorder.
